# Comparison of a direct aspiration first pass technique vs. stent retriever thrombectomy for the treatment of acute large vessel occlusion stroke in the anterior circulation with atrial fibrillation

**DOI:** 10.3389/fneur.2023.1138993

**Published:** 2023-02-24

**Authors:** Hongxing Fan, Zhenhui Li, Yi Li, Yanping Tan, Zhenlin Mao, Qian Liu, Youfeng Zhu

**Affiliations:** ^1^Department of Neurology, Guangzhou Red Cross Hospital of Jinan University, Guangzhou, China; ^2^Department of Critical Care Medicine, Guangzhou Red Cross Hospital of Jinan University, Guangzhou, China

**Keywords:** acute large vessel occlusion stroke, atrial fibrillation, mechanical thrombectomy, stent retriever thrombectomy, a direct aspiration first pass technique

## Abstract

**Objectives:**

The stent retriever thrombectomy (SRT) and a direct aspiration first-pass technique (ADAPT) are the two main mechanical thrombectomy (MT) techniques for acute ischemic stroke. Few data are available for comparing the therapeutic effects associated with the two mechanical thrombectomy techniques in acute ischemic stroke with atrial fibrillation. The purpose of this study was to compare the efficacy and safety of both techniques for the treatment of acute large vessel occlusion stroke in the anterior circulation with atrial fibrillation.

**Methods:**

Retrospective analysis was performed in stroke patients with atrial fibrillation admitted to Guangzhou Red Cross Hospital from January 2018 to June 2022 who received mechanical thrombectomy by either SRT or ADAPT. Comparisons were made with regards to the initial traits, course of therapy, effectiveness indicators, and complications of these individuals. The primary outcome is recanalization rate.

**Results:**

In this study, after screening 431 patients, 92 eligible patients, with 48 patients received SRT and 44 patients received ADAPT, were included. There was no significant difference in the recanalization rate between the two groups (SRT 87.5% vs. ADAPT 84.1%, *P* = 0.639). Compared with SRT, patients in ADAPT group had a shorter puncture to recanalization time [33.5 min (27.0–59.5) vs. 50.5 min (31.5–91.5), *P* = 0.009], a higher first pass success recanalization rate (54.5 vs. 33.3%, *p* = 0.040), and a higher rate of patients with improvement of NIHSS scores ≥4 at discharge (84.1 vs. 56.3%, *P* = 0.004). However, distal embolization occurred more frequently in the ADAPT group than that in SRT group (50.0 vs. 22.9%, *P* = 0.007). There was no significant difference between the two groups in the 3-month mRS score, symptomatic cerebral hemorrhage, or mortality.

**Conclusions:**

Compared with SRT, ADAPT has similar recanalization rate for the treatment of acute large vessel occlusion stroke in the anterior circulation with atrial fibrillation. However, ADAPT might be more effective in terms of shorter puncture to recanalization time and higher first pass success recanalization rate. Further studies are needed for confirming our results.

## Introduction

Atrial fibrillation (AF) is an important risk factor for ischemic stroke (1–4). Acute ischemic stroke in patients with AF is associated with a higher degree of disability and worse outcomes than that in patients without AF (5–7). Most stroke patients with AF have large vessel lesions, and treatment with intravenous thrombolysis alone is less effective in terms of both recanalization and clinical outcome (8, 9). Mechanical thrombectomy has become the standard of treatment in patients with acute ischemic stroke with large vessel occlusion. SRT and ADAPT are the two main mechanical thrombectomy procedures, which are comparable in thrombectomy efficiency (10, 11). However, these previous studies focused on the efficacy of devices for thrombectomy in specific intracranial vessels and specific anatomical sites, neglected to take into account the characteristics of embolus features on the efficacy of thrombectomy. Atrial fibrillation is the main cause of cardiogenic stroke. Most cardiogenic emboli are old emboli containing a high percentage of white blood cells and are characterized by a large shape, hard texture and relative integrity (12–14). Based on limited previous clinical observations, different methods of mechanical thrombectomy may have different re-canalizations for stroke patients with thrombi of specific properties.

The purpose of this study is to compare the efficacy and safety of SRT and ADAPT for the treatment of acute large vessel occlusion stroke in the anterior circulation with atrial fibrillation.

## Methods

### Study design

This is a single center retrospective study in the Neurology Department of a tertiary university hospital (Guangzhou Red Cross Hospital of Jinan University). Our study has received approval from the ethics committee of our hospital (approval number 2022-280-01).

### Patients

This study involved patients who underwent mechanical thrombectomy (MT) for acute ischemic stroke at Guangzhou Red Cross Hospital from January 2019 to June 2022. These individuals had an AF diagnosis before admission or an ECG examination proving AF prior discharge and fulfilled the following criteria: (1) age >18 years, (2) symptom onset < 6 h, (3) an Alberta Stroke Program Early CT Score (ASPECTS) >6, 4) a National Institute of Health Stroke Scale (NIHSS) score ≥5, (5) independent daily living (mRS < 3) before the index stroke, (6) DSA confirmed large vessel occlusion in anterior circulation.

### Endovascular treatment

If patients were eligible for intravenous thrombolysis, 0.9 mg/kg recombinant tissue-type fibrinogen activator (rt-PA) was administered before mechanical thrombectomy according to Chinese guidelines for the endovascular treatment of acute ischemic stroke (15). The mechanical thrombectomy procedure was performed by two interventional neuroradiologists with 10 years of practice in neurointerventions. The choice of STR or ADAPT was left to the discretion of the operator, usually based on the anatomical location of the thrombus obstruction, preoperative judgment of the etiology and pathogenesis, and the size of the thrombus. All patients were treated with local anesthesia, preferably through the right femoral artery, to establish access. A balloon guide catheter (BGC) was not used in all procedures due to limitations in available device conditions. The ADAPT and SRT techniques have been described previously (16, 17). Patients received ADAPT using AXS Catalyst-6 (Stryker, USA) as front-line therapy. All stent retriever procedures were performed using the Solitaire FR (Covidien, USA). Meanwhile, intermediate catheters (AXS Catalyst-6) were routinely used. The operator could choose any necessary thrombectomy device and method to obtain an acceptable therapeutic effect if a successful recanalization could not be accomplished after three attempts using SRT or ADAPT.

### Data collection

The patient baseline characteristics, the anatomical structure of vascular access, medical history, procedure-related time points, stroke severity, recanalization devices, number of mechanical thrombectomy attempts, need for rescue therapy and 3-month follow-up data were collected. The degree of vessel occlusion before and after treatment was defined by the extended Thrombolysis in Cerebral Infarction (eTICI) classification, and a postoperative eTICI score 2c/3 was defined as successful recanalization of the vessel. The NIHSS score was used to determine the level of neurological severity (ranges from 0 to 42, with higher scores indicating a greater degree of severity), and improvements of at least 4 points on the NIHSS score within 24 h or at discharge than that of admission were considered short-term neurological improvement. The modified Rankin Scale (mRS) was used to assess neurological recovery at 90 days postoperative, and mRS score of 0 to 2 was defined as favorable neurological recovery. All patients were reexamined by head CT or MRI scan within 24 h after the operation to determine whether intracranial hemorrhage occurred, and symptomatic intracerebral hemorrhage (sICH) was defined as the presence of hemorrhage after the procedure, with worsening of clinical examination by ≥4 points on the NIHSS.

### Primary and secondary outcomes

The primary outcome is recanalization rate. The secondary outcomes are improvement of NIHSS score at 24 h after procedure and discharge, 90-day favorable clinical prognosis, symptomatic intracranial hemorrhage and 90-day all-cause mortality.

### Statistical analysis

Statistical analysis was performed using SPSS 23.0 software (IBM Corp., NY, USA). Measurement data obeying a normal distribution were expressed as the mean ± standard deviation (SD), the median (interquartile range) was used for data not obeying a normal distribution, and an independent sample *t*-test or one-way analysis of variance was used for comparison between groups. Categorical variables were expressed as percentages, and the Fisher's exact test was used for comparison between groups. Two-sided *P*-values were taken for all tests, and *P* < 0.05 was deemed statistically significant.

## Results

**Figure 1:**
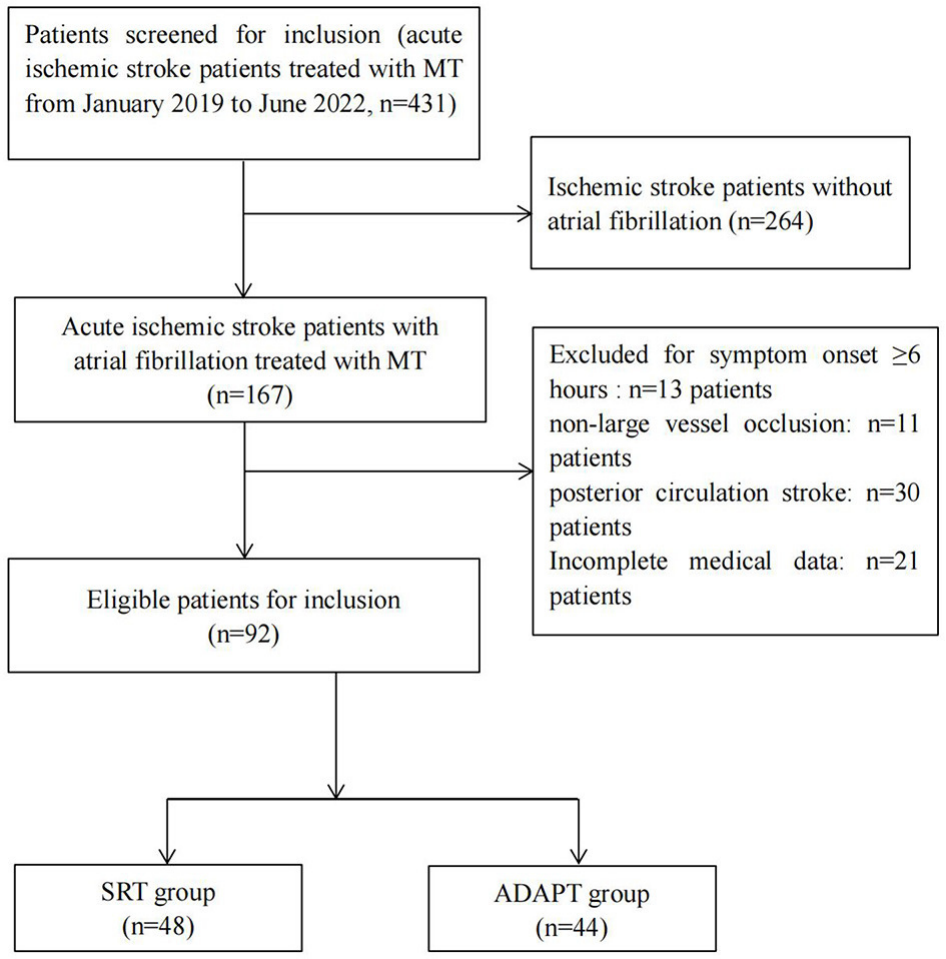
Flowchart of patients inclusion process. MT, mechanical thrombectomy; SRT, stent retriever thrombectomy; ADPAT, a direct aspiration first-pass technique.

From January 2019 to June 2022, 431 patients admitted to the Neurology Department of Guangzhou Red Cross Hospital were screened for eligibility. Of these patients, 92 were eligible, and were included in the study. Forty-eight patients underwent SRT and 44 patients underwent ADAPT approach (Figure 1). Patients' baseline characteristics are shown in Table 1. The patients were 71.5 (68.0–85.5) years old in the SRT group (female 54.2%) and 75.5 (69.0–84.0) years in the ADAPT group (female 43.2%). There were no significant differences in baseline characteristics (age, gender, baseline NIHSS score, ASPECTS, intravenous thrombolysis, occlusion location, the presence of internal carotid artery (ICA) tortuosity, risk factors, onset to puncture time and door to puncture time) between the two groups.

**Table 1:** Baseline characteristics of the stroke patients with AF treated by ADAPT or SRT.

		**SRT (*n* = 48)**	**ADAPT (*n* = 44)**	***P***
Age, median (IQR)		71.5 (68.0–85.5)	75.5 (69.0–84.0)	0.836
Gender (female), *n* (%)		26 (54.2)	19 (43.2)	0.292
NIHSS at baseline, median (IQR)		15 (10.5–20.5)	12 (10–19)	0.242
ASPECTS at baseline, median (IQR)		10 (9–10)	10 (8.5–10)	0.55
Prior IVT, *n* (%)		18 (37.5)	18 (40.9)	0.738
**Risk factors**, ***n*** **(%)**				
	Hypertension	34 (70.8)	30 (68.2)	0.782
	Diabetes mellitus	8 (16.7)	7 (15.9)	0.922
	Hyperlipidemia	18 (37.5)	9 (20.5)	0.073
	Coronary artery disease	18 (37.5)	15 (34.1)	0.733
	Prebious stroke	8 (14.6)	6 (13.6)	0.896
	Smoking	14 (29.2)	11 (25.0)	0.654
**Occlusion location**, ***n*** **(%)**				
	ICA	9 (18.8)	15 (34.1)	0.094
	M1	31 (64.6)	25 (56.8)	0.446
	M2	3 (6.3)	2 (4.5)	0.719
	A1/A2	5 (10.4)	3 (6.8)	0.541
ICA tortuosity, *n* (%)		30 (62.5)	27 (61.4)	0.911
**Timing (min), median (IQR)**				
	Onset to puncture	209.5 (140.5–374.5)	174.5 (115–413)	0.334
	Door to puncture	92 (64.5–116)	74 (59.5–102)	0.134
Puncture to recanalization (min), median (IQR)		50.5 (31.5–91.5)	33.5 (27.0–59.5)	0.009
Remedy with devices, *n* (%)		17 (35.4)	22 (50.0)	0.157
First pass success, *n* (%)		16 (33.3)	24 (54.5)	0.040
Final eTICI 2c/3, *n* (%)		42 (87.5)	37 (84.1)	0.639
Distal embolization, *n* (%)		11 (22.9)	22 (50.0)	0.007

IQR, interquartile range; NIHSS, National Institutes of Health Stroke Scale; ASPECTS, Alberta Stroke Program Early CT Score; IVT, intravenous thrombolysis; ICA, internal carotid artery; M1/2, middle cerebral artery segments; A1/A2, anterior cerebral artery segments; AF, atrial fibrillation; eTICI, expanded Thrombolysis In Cerebral Infarction score.

There was no significant difference in the final recanalization rate (eTICI 2c/3) between the two groups (SRT 87.5% vs. ADAPT 84.1%, *P* = 0.639). The ADAPT group had a higher first-pass successful recanalization rate than that in the SRT group (54.5 vs. 33.3%, *p* = 0.040). Compared with the SRT group, the puncture to recanalization time was shorter in the ADAPT group [50.5 min (31.5–91.5) vs. 33.5 min (27.0–59.5), *p* = 0.009]. Distal embolization occurred more frequently in the ADAPT group (ADAPT 50.0% vs. SRT 22.9%, *P* = 0.007). However, there were no significant differences with regards to the use of additional devices between the two groups (ADAPT 50.0% vs. SRT 35.4%, *P* = 0.157) (Table 1).

There were no statistically significant differences with regards to the favorable outcome (mRS 0–2), symptomatic intracranial hemorrhage, or mortality at 3-month between the two groups. However, compared with SRT group, more patients in ADAPT group demonstrated a higher ratio of an improvement of NHISS scores ≥4 at discharge (ADAPT 84.1 vs. SRT 56.3%, *p* = 0.004). Additionally, the total medical cost in the ADAPT group was significantly lower than that in the SRT group [$14038.6 (11330.9–17367.3) vs. $16450.9 (14307.7–18923.1), *p* = 0.014]. A shorter hospital stay was observed in the ADAPT group, but this did not reach statistical significance (Table 2).

**Table 2:** Clinical outcomes in stroke patients with AF treated by ADAPT or SRT.

	**SRT (*n* = 48)**	**ADAPT (*n* = 44)**	***P***
Improvement of NHISS scores ≥4 at 24 h, *n* (%)	24 (50.0)	28 (63.6)	0.188
Improvement of NHISS scores ≥4 at discharge, *n* (%)	27 (56.3)	37 (84.1)	0.004
90-d mRS 0–2, *n* (%)	20 (41.7)	24 (54.5)	0.217
sICH, *n* (%)	5 (10.4)	2 (4.5)	0.289
90-d Mortality, *n* (%)	9 (18.8)	5 (11.4)	0.324
Length of stay (d), median (IQR)	10 (9.0–12.0)	9 (7.5–11.0)	0.246
Hospital charges (¥), median (IQR)	111,456.0 (97,924.3–129,805.2)	95,112.6 (76,923.5–116,552.0)	0.014

NIHSS, National Institutes of Health Stroke Scale; mRS, modified Rankin scale; sICH, symptomatic intracranial hemorrhage; IQR, interquartile range.

## Discussion

The results of the study indicated that there was no significant difference between SRT and ADAPT for the treatment of acute large vessel occlusion in the anterior circulation with atrial fibrillation in terms of final recanalization rate, 90-day favorable clinical prognosis, symptomatic intracranial hemorrhage, and 90-day all-cause mortality. To our knowledge, this is the first study to specifically investigate SRT and ADAPT in stroke patients with atrial fibrillation.

Atrial fibrillation is the primary cause of cardiogenic stroke. Cardiogenic emboli have distinct properties. Most cardiogenic emboli are old emboli, which are characterized by a large size, hard texture, complete shape, and relatively free relationship with the vessel wall (12, 13). Neurointerventionists are concerned about whether these characteristics affect the effectiveness of mechanical thrombectomy in acute ischemic stroke. This study showed no significant difference in achieving favorable recanalization (eTICI2c/3) between the ADAPT and SRT in stroke patients with atrial fibrillation, which was consistent with previous findings in other types of stroke (11, 17–20). However, several studies contend that aspiration thrombectomy for ischemic stroke with intracranial large vessel occlusion results in a higher rate of recanalization, especially in posterior circulation lesions where the advantages of aspiration thrombectomy are more prominent (21, 22). In this study, the ADAPT group showed a higher first-pass successful recanalization rate and a shorter time from femoral artery puncture to successful reperfusion, suggesting that the ADAPT technique is more efficient for thrombectomy in atrial fibrillation stroke than SRT. One rationale for the remarkable effectiveness of the aspiration approach for thrombectomy in atrial fibrillation stroke is that the aspiration catheter depends on strong negative pressure after contact and can remove the entire thrombus at once.

According to the previous study, the anatomical structure of the anterior circulation are important factors related to the recanalization rate and duration of first-time recanalization. The incidence of carotid artery tortuosity is high in stroke patients with atrial fibrillation (23). Therefore, the anatomy of vascular access may influence the findings. However, no significant difference was found in our observations with regard to this aspect.

Distal embolization is associated with worse clinical outcomes (24–27) and is currently a growing concern. In this study, we discovered that the presence of distal emboli was significantly higher in the ADAPT group than in the stent retriever group. The internal diameter of the aspiration catheter is an essential factor in determining the efficiency of thrombectomy and the need for supplementary treatment (28). The aspiration catheter can remove the entire thrombus at once if the embolus volume is less than or equal to the inner diameter of the aspiration catheter. However, if the embolus volume is greater than the inner diameter of the aspiration catheter, aspiration therapy will damage the integrity of the embolus and cause distal secondary emboli. With an inner diameter of 0.068 inches, the AXS Catalyst-6 catheter was used in this study. In some cases, larger emboli could not be aspirated into the lumen of AXS Catalyst-6, and negative pressure instead caused fragmentation or residual emboli, and small emboli escaped distally after blood flow was restored. The texture of cardiogenic emboli is hard and fragile, which may be another cause of distal emboli. Future aspiration catheter performance improvements and catheter lumen expansions may enhance aspiration thrombectomy effectiveness and reduce distal emboli in stroke patients with atrial fibrillation.

Early and efficient revascularization of large vessel occlusions has been shown to correlate with improved outcomes in patients with acute ischemic stroke (29, 30). In this study, the ADAPT group had a shorter time from femoral artery puncture to successful recanalization and a greater improvement of NHISS scores ≥4 at discharge than the stent retriever group. Meanwhile, the rates of improvement of NHISS scores ≥4 at 24 h after procedure and the rates of good neurological outcome (mRS 0–2) at 90 days showing a trend toward higher improvement have also been observed in the ADAPT group, although they did not reach statistical significance, which was thought to be caused by a higher rate of distal embolization partially offsetting the clinical benefit from aspiration efficiency in the ADAPT group.

Total costs during hospitalization were lower in the ADAPT group in our research, which is consistent with earlier findings (16, 31). In addition to saving the cost of stent during the procedure, shorter hospital stays (although not statistically significant) were also a contributing factor in reduced total hospital expenditures. The ADAPT approach is more advantageous for the promotion of this technique in clinical practice and has lower health care costs under the assumption of equivalent thrombectomy efficiency.

## Conclusion

The results of this study reveal that ADAPT and SRT have similar final recanalization rate for the treatment of acute large vessel occlusion stroke in the anterior circulation with atrial fibrillation. However, ADAPT might be more effective in terms of shorter puncture to recanalization time and higher first pass success. The findings offer some theoretical support for choosing mechanical thrombectomy for this kind of stroke. However, there are some shortcomings in this study. First, The AXS Catalyst-6 thrombus aspiration catheter, used uniformly in this study, had a smaller internal diameter than other similar products, which had an impact on the study results. Second, according to the previous study, the presence or absence and induction of BGC are important factors related to the recanalization rate and duration of first-time recanalization (23, 32). However, A BGC was not used in all procedures due to limitations in available device conditions which may influence our results. Third, it is a single-center retrospective study with a limited sample size. The choice of STR or ADAPT was based on operator's discretion. The results of this study may be biased. In the future, with the advancement of interventional materials, multicenter, prospective, randomized controlled clinical investigations are needed to further validate the study results.

## Data availability statement

The raw data supporting the conclusions of this article will be made available by the authors, without undue reservation.

## Ethics statement

The studies involving human participants were reviewed and approved by the Ethics Committee of Guangzhou Red Cross Hospital (approval number 2022-280-01). Written informed consent for participation was not required for this study in accordance with the national legislation and the institutional requirements.

## Author contributions

HF and YZ were responsible for the study of design, data analysis, and drafting of the manuscript. HF and ZL were responsible for performing mechanical thrombectomy procedure and patients' care. YL, ZM, YT, and QL took part in patient care, patients' follow-up, and data collection. All authors contributed to the article and approved the submitted version.
